# Relationships Between Self-Efficacy, Self-Esteem and Procrastination in Undergraduate Psychology Students

**Published:** 2014

**Authors:** Nader Hajloo

**Affiliations:** 1 Department of Psychology, School of Education Sciences and Psychology, University of Mohaghegh Ardabili, Ardabil, Iran.

**Keywords:** Mediation, Procrastination, Self-Efficacy, Self-Esteem

## Abstract

**Objective:** The present study aimed to review the relationships between procrastination and two self-factors self-efficacy and self-esteem.

**Methods:** Participants were 140 undergraduates Psychology students enrolled in Mohagheg Ardabili University, Ardabil, Iran. Instruments used for collecting the required data were the student-version of the General Procrastination Scale (GP-S), General Self-Efficacy Scale (GSE) and Rosenberg’s Self-Esteem Scale (SES).

**Results: **Using causal modeling, two models were compared; a model with self-esteem as a mediator versus a model with procrastination as a mediator. The self-esteem mediator model accounted for 21% of the variance in procrastination. The significance of the mediation effect was found by bootstrapping method.

**Conclusion:** The relationship of procrastination with self-esteem and self-efficacy was revealed among undergraduate psychology students.

## Introduction

Procrastination behavior is very common and is a serious problem in our world. However it seems that researchers cannot reach a consensus on the definition of this phenomenon (1). Intentional delaying in doing something is proposed as the definition of procrastination (1, 2). According to the studies regarding tendency to procrastination, the reasons listed are poor time management skills, self-efficacy (SEF) beliefs, self-esteem, discomfort regarding tasks, personal characteristics (responsibility, perfectionism, neurotic tendency, etc), irrational thoughts, inability to concentrate, fear of failure, inability to orient objectives of success, lowered self-respect, anxiety, problem-solving skills, unrealistic expectations, and working habits (3-10). It is assumed that procrastination is related to low self-esteem, either as a determinant or a consequence. However, the negative correlation is assumed to be between self-esteem and procrastination (11).

SEF theory (12) holds that what we believe about ourselves strongly influences our task choice, level of effort and persistence, and how we subsequently perform. Bandura argued that if adequate levels of ability and motivation exist, initial attempts to do and continue to work, will be affected by SEF. Weak poor efficacy may be involved in avoidance behavior but strong SEF may play a role in the onset and persistence of behavior (13). SEF has been found to be one of the strongest factors predicting performance in various domains. In academic settings, SEF is a strong predictor of performance, with the strength of association dependent upon correspondence with the task in question, as well as level of specificity (14). SEF has been studied in several previous procrastination studies, with results showing an inverse relationship with procrastination (2, 15-18).

Another construct that is often connected witch procrastination, self-esteem, refers to judgments of global self-worth (12). According to Tesser (19), “SES is a global evaluation reflecting our view of our accomplishments and capabilities, our values, our bodies, other’s responses to us, and events, or occasions, our possessions”. The relationship between procrastination and self-esteem has received considerable attention in the procrastination literature. Procrastination has been described as a self-protective strategy that masks a fragile self-esteem, and numerous studies have found a significant inverse relationship between self-report procrastination and self-esteem (20-23). Flett et al. (24) proposed that procrastinators suffer from low self-esteem that results in a general tendency to engage in behaviors -like task delay and avoidance- that protect self-presentation by providing an excuse for poor performance and negative outcomes. Although the most recent procrastination studies explain procrastination through SEF or self-regulation models (2, 7, 18), considerable research has explored and continues to explore the link between procrastination and self-esteem (25).

Self-esteem and SEF appear to be very different constructs (26). Questions of SEF are related to one's ability to perform certain tasks or actions**,** the outcomes of which may or may not have any bearing on self-esteem. Thus, if an individual has high levels of SEF on tasks within an occupation in which he/she has invested much self-worth then there is likely to be a positive correlation between SES and SEF (12).

Regarding the relationship of SEF and SES, Stroiney (27) suggested that high SEF is predictive of high SES; whereas, low SEF predicts low self-esteem. As Bandura (12) points out “self liking does not necessarily beget performance attainments”. Research findings demonstrate that self-esteem predicts neither the choice of personal goals, nor performance accomplishments (28).

Therefore, it can be inferred that SEF predicts self-esteem (rather than self-esteem predicts SEF), particularly in predicting trait procrastination. Individuals with low SEF may be more likely to delay in decision making. One critical issue centers on the presumed orthogonal nature of SEF and self-esteem. Specifically, some people may question whether it is conceptually sound to assume that the two orthogonal variables can predict each other; they might assert that the relationship of SEF and self-esteem would be best described by a simple, reciprocal correlation/covariance. Then this relation between variables might result from their shared variability (or error variance). However, we argue that this issue remains unresolved. 

The purpose of this study was to determine the relationship between the quality of procrastination with SEF and self-esteem. This study would answer two basic questions by comparison two models of mediation: first, which variable (procrastination, SEF, or self-esteem) is a mediator. Second, is the mediation effect statistically important?

## Materials and Methods

***Participants***

Participants were 140 (male, n = 52; female, n = 88) undergraduates Psychology students enrolled in Mohagheg Ardabili University, Ardebil, Iran. Participants’ mean age was 20.5 years (±SD = 3.8). The students filled out a number of research instruments over a semester period. 

***Instruments***

*The Student-version of the General Procrastination Scale* *(GP-S) *(29)

General Procrastination Scale contains 20 items on 5-degree Likert scale to measure procrastination. *Alpha* value (α) for the GP-S is reported to be 0.90 in Lay study (30), and 0.85 in Lay and Silverman study (31). In the present study, *α* value acquired was 0.83. Retest reliability of this scale has been reported to be appropriate (32). In addition, its validity has been confirmed in previous reports (33, 34). 

*The* *General Self-Efficacy Scale* *(* *GSE* *)*

The scale in German Language was developed by Schwarzer and Jerusalem (35) and later was revised and adapted to 26 other languages including English and Persian. This scale is made for people aged 12 and older. The scale has 10 items with 4 point scale, ranging from 1 to 4 (1 = not at all true), (2 = hardly true), (3 = moderately true), to (4 = exactly true). Responses to all 10 items have to be summed up to yield the final composite score with a range from 10 to 40. In studies over 23 nations, Cronbach's alpha values were between 0.76 and 0.90. The scale is one-dimensional. Criterion validity of this scale has been noted to be appropriate. The scale has shown positive relationship with positive emotions and negative relationship with mental health problems.

*Self-Esteem Scale (* *SES* *)*

This scale is made by Rosenberg based on Gatman scale (36). The scale consists of 10 one-dimensional items on 4 point scale (1 = strongly disagree to 4 = strongly agree) and there is a balance between positive and negative items. Scoring of the 5 items is reveres. Total score of SES is obtained from the sum of subject responses to all items of the scale. Scores range is between 10-40 and the highest score indicating the highest level of self-esteem. In previous studies,* Alpha* value (α) for the scale has been reported as 0.82 (26) and in the current study this value was obtained as 0.85.

## Results

***Descriptive statistics***

Of the total 140 students who contributed in this study, 132 students (83 females and 49 males) completed the GP-S, GSE and SES and were included in the final statistical analyses. Descriptive statistics for procrastination, SEF, and SES are presented in table 1. As expected, procrastination was negatively correlated with SEF (r = -0.32, p < 0.01), indicating that higher procrastination level was associated with lower SEF, and negatively correlated with SES (r = -0.29; 0 < 0.01), indicating that higher procrastination was related to lower SES. In addition, SEF and SES were positively correlated (r = 0.37; p < 0.01), suggesting that higher levels of SES were associated with higher levels of SEF.

***Testing mediation effects***

Data were analyzed with AMOS (Asset Management Operating System) software for Windows 16.0. First the measure, then the three structural models were evaluated (37): 1) SES will mediate the relationship between the SEF and procrastination, 2) SEF will mediate the relationship between the SES and procrastination, and 3) Procrastination will mediate the relationship between the SES and SEF. The fit indices of models were compared with Holmbeck's procedures (38) and the statistical significance of mediation role was evaluated with bootstrapping procedures (39). To control the measurement errors inflammation caused by using more items, 9 item parcels were created (40, 41): two parcels for both SEF and SES (five items per one parcel) and five parcels for procrastination (four items per one parcel).

***Measurement model***

A lambda for each latent variable in the measurement model (SEF, SES, and procrastination), were bound to 1, but the parameters of the three routes between the latent variables were freely estimated. To test the significance of the measurement model, following criteria were used (40, 42): χ^2^, SRMR (standardized root-mean-square residual), CFI (comparative fit index), RMSEA (root-mean-square error of approximation), AIC (Akaike information criterion), and ECVI (expected cross-validation index). Measurement model of this study showed significant: χ^2 ^(20, N = 132) = 51.02, p = 0.021; RMSEA = 0.03 (90% CI: 0.01-0.05); SRMR = 0.03, CFI = 0.99, AIC = 109.62, and ECVI = 0.35 (CI: 0.29-0.42). Then, the structural model can be assessed.

**Table 1:** Descriptive statistics and inter-correlations

	**1**	**2**	**3**	**M**	**SD**
**1. Procrastination** †	-			57.33	12.91
**2. Self-efficacy**	-0.32	-		29.12	06.35
**3. Self-esteem**	-0.29	0.37	-	33.10	04.08

Note. Number of students = 132.

Self-efficacy and Self-esteem are two variables of self cognition. All correlation coefficients were significant at p < 0.01.

† A student version of general procrastination scale (GP-S)

**Table 2:** Structural paths, chi-square, and fit indices among different models

	**SEF** † ** → SES** ‡ ** → P** §		**SES** ‡ ** → SEF** † ** → P** §		**SEF** † ** → P** § ** → SES** ‡	
	**Model 1-1** **(Full)**	**Model 1-2 (Partial)**	**Model 2-1 ** **(Full)**	**Model 2-2 (Partial)**	**Model 3-1 ** **(Full)**	**Model 3-2 (Partial)**
**SES** ‡ ** → P**	-0.53	-0.52	-0.52			
**SES** ‡ ** → SEF** †			0.39	0.44		
**SEF** † ** → SES** ‡	0.39	0.39			0.08	
**SEF** † ** → P**	0.07		0.07	-0.36	0.41	-0.43
**P** § ** → SES** ‡					-0.37	-0.34
**χ** ***2***	51.02	52.44	51.02	196.23	51.02	56.21
**df**	21	20	21	20	21	20
**RMSEA** ||	0.03	0.03	0.03	0.09	0.03	0.04
**CI** ¶ ** for RMSEA**	0.01–0.05	0.01-0.05	0.01–0.05	0.08-0.11	0.01–0.05	0.02-0.06
**SRMR** ††	0.03	0.03	0.03	0.17	0.03	0.04
**CFI** ‡‡	0.99	0.99	0.99	0.89	0.99	0.98
**AIC** §§	109.62	108.49	109.62	248.14	109.62	130.51
**ECVI** ‡‡‡	0.35	0.35	0.35	0.80	0.35	0.36
**CI for ECVI**	0.29–0.42	0.29-0.42	0.29–0.42	0.68-0.97	0.29–0.42	0.30-0.44

Note. N = 132.

† = Self-efficacy;

‡ = Self-esteem;

§ = Procrastination. Boldface type represents the best model; dashes indicate paths that were constrained to zero;

|| = root-mean-square error of approximation;

¶ = confidence interval;

†† = standardized root-mean-square residual;

‡‡ = comparative fit index;

§§ = Akaike information criterion; and

‡‡‡ = expected cross-validation index. All chi-square values were significant at p < 0.001.

***Structural models***

*The SES mediator model (Model 1):* Direct path between the self-efficacy and procrastination was tested: the path coefficient was significant, B = 0.26, p < 0.01. Next, a partially-mediated model (Model 1-2) was tested by adding both paths from SEF to SES and from SES to procrastination. This model revealed a good fit to the data: χ^2 ^(20, N = 132) = 51.02, p = 0.021; RMSEA = 0.03 (90% CI = 0.01-0.05); SRMR = 0.03, CFI = 0.99, AIC = 109.62, and ECVI = 0.35 (CI: 0.29-0.42). However, the direct path coefficient from SEF to procrastination (B = -0.07) was not statistically significant, which supported a fully-mediated model with this path constrained to zero. The results for the SES full mediator model showed a very good fit: χ^2 ^(21, N = 132) = 52.44, p = 0.018; RMSEA = 0.03 (90% CI = 0.01-0.05); SRMR = 0.03, CFI = 0.99, AIC = 108.49, and ECVI = 0.35 (CI: 0.29-0.42). Taken together, for the SES mediator model, the full mediator model (Model 1-1) was selected over the partial mediator model (Model 1-2) (Table 2).

***The SEF mediator model (Model 2):***

The SEF partial mediator model (Model 2-2) was not supported because the direct path from SEF to procrastination was not significant, B = -0.07; p > 0.05. For the SEF full mediator model (Model 2-1), the fit indices were poor: χ^2 ^(21, N = 132) = 196.23, p = 0.001; RMSEA = 0.09 (90% CI = 0.08-0.11); SRMR = 0.17, CFI = 0.89, AIC = 248.14; and ECVI = 0.80 (CI: 0.68-0.97). 

***The procrastination mediator model (Model 3)***

Similar procedures were employed to compare the procrastination full mediator model (Model 3-1) with the procrastination partial mediator model (Model 3-2). First, the direct path from SEF to SES was significant, B = 0.29, p < 0.01. The procrastination partial mediator model revealed a good fit to the data: χ^2 ^(20, N = 132) = 51.02, p = 0.021; RMSEA = 0.03 (90% CI = 0.01-0.05); SRMR = 0.03, CFI = 0.99, AIC = 109.62, and ECVI = 0.35 (CI: 0.29-0.42). However, the direct path coefficient from SEF to SES (B = 0.08) was not statistically significant, which supported a fully-mediated model with this path constrained to zero. The results for the procrastination full mediator model showed a very good fit: χ^2 ^(21, N = 132) = 56.21, p = 0.010; RMSEA = 0.04 (90% CI = 0.02-0.06); SRMR = 0.04, CFI = 0.98, AIC = 130.51, and ECVI = 0.36 (CI: 0.30–0.44). Taken together, for the procrastination mediator model, the full mediator model (Model 3-1) was selected over the partial mediator model (Model 3-2) (Table 2). Generally, the study findings supported the SES full mediator model over the SEF and procrastination full mediator models.

***Analyses of the mediator effect in model 1-1using bootstrap***

**Figure 1:**
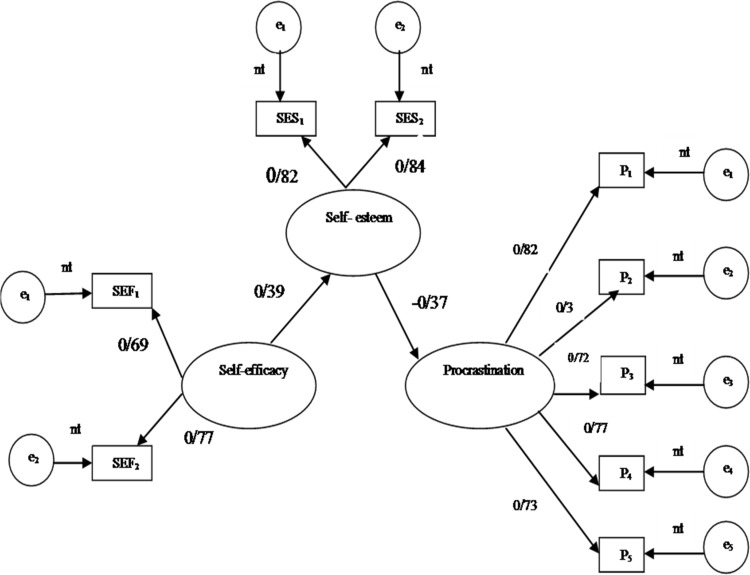
The self-esteem full meditor model. Note. The rectangles are measured variables, the large circles are latent constructs, and the small circles are residual variances. Factor loadings are standardizied and all are significant (p < 0.001) except for those designated paths, nt = fixed to1. SEF1-SEF2 = two parcels from the Self-efficacy; SES1-SES2 = two parcels from the Self-esteem, and P1-P5 = five parcels from the general procrasination scale. N = 132.

To use the bootstrapping, beginning the 1000 samples were extracted from the research data (N = 132). This sampling method was replaced. Then, using the sub-samples, Model 1-1 was evaluated 1,000 times (43). Finally, by multiplying 1,000 dual path coefficients, estimating mediated effect was obtained. If the zero was not included in the 95% CI, indicates that the mediator effect is significance at 0.05 level (39). The results of bootstrapping revealed that the effect of mediator from SEF through SES to P (β = 0.19 [CI: 0.10, 0.31]) was statistically significant. The amount of the mediator effect was B = -0.53 × 0.37 = -0.21, which indicated that 21% of the variability in procrastination trait was explained by the mediator effect in Model 1-1 (Figure 1).

## Discussion

The results showed that self-esteem full mediator model in front of the alternative models was accepted, highlights Flett et al. (24) research that self-esteem is likely to influence procrastination. The mediation model suggests that procrastinators suffer from low SES that result in a general tendency to engage in behaviors, like task delay and avoidance, that protect self-presentation by providing an excuse for poor performance and negative outcomes. Thus, counselors/educators should target procrastinators' level of self-esteem, in addition to their levels of self-efficacy. Helping students reinforce self-efficacy may lower procrastinating tendencies; however, our findings indicated that intervention could be more effective if students are assisted in raising their levels of self-esteem. If self-esteem refers to judgments of global self-worth (12), interventions targeted to develop students’ problem-focused coping strategies may increase their motivation levels, control their self-esteem, identify and approach problematic situations with specific goals, and generate alternative solutions. On the other hand, to help people who are often reluctant to intern and procrastinator, can be used to teach problem-solving (44).

In this study, SEF weakly mediated relationship between SES and procrastination, this result supports the SES full mediator model. So, although there is a moderate relationship between SES and SEF (12), the SES can better predict the procrastination.

Regarding the relationship of self-efficacy and self-esteem, Stroiney, (27) suggested that high self-efficacy is predictive of high self-esteem; whereas, low self-efficacy predicts low self-esteem. Therefore, it can be inferred that self-efficacy predicts self-esteem (rather than self-esteem predicts self-efficacy), particularly in predicting procrastination.

The results of this study can be best understood within a coping and problem-solving framework. In this way, the suggestions of previous researchers (45) in helping to reduce procrastination can be effective.

There were several limitations in this study: 1) The results of this research can be extended to society that sample was derived from it. 2) In this study, possible differences between procrastinators were not studied. Therefore, it is not clear whether by intensity the procrastination, what changes occur in the mediation model (46). 3) Type of this research was correlation, so causation cannot be concluded. In order to achieve this goal, longitudinal studies are needed. 

Regardless of these limitations, this study revealed the relationship of procrastination with self-esteem and self-efficacy among undergraduate psychology students. This work is the first research that tried to evaluate the mediator effect of self-esteem in the relationship between self-efficacy and procrastination. 

## Notes

**Declaration of interest:** None.
